# Mandating doctors to attend counter-terrorism workshops is medically unethical

**DOI:** 10.1192/pb.bp.115.053173

**Published:** 2016-04

**Authors:** Derek Summerfield

**Affiliations:** 1 South London and Maudsley NHS Foundation Trust, London, UK

## Abstract

This is a brief exploration of the ethical issues raised for psychiatrists, and for universities, schools and wider society, by the demand that they attend mandatory training as part of the UK government's Prevent counter-terrorism strategy. The silence on this matter to date on the part of the General Medical Council, medical Royal Colleges, and the British Medical Association is a failure of ethical leadership. There is also a civil liberties issue, reminiscent of the McCarthyism of 1950s USA. We should refuse to attend.

I want to raise a matter of immediate ethical import for doctors, one which does not seem to have been debated in medical journals. My National Health Service (NHS) trust told us recently that it is mandatory for all staff to attend a Workshop to Raise Awareness of Prevent, part of the government's so-called Prevent counter-terrorism strategy. This is so across the whole NHS, and tens of thousands have apparently attended already. The workshops intend to offer guidance on how to identify people who may be vulnerable to ‘radicalisation’ and on how to refer them on. This is a corrosion of the ethics of the doctor-patient relationship, and is to prime us for an activity which is a duplicitous deviation from the medical assessment, advice and treatment that has brought the patient to us. It is basically a form of spying and of scapegoating, and essentially about Muslim patients. An example provided by a psychiatrist on the Critical Psychiatry Network recently was of a young Muslim man with a mild depressive picture who was referred by a general practitioner (GP) for a psychiatric assessment to explore his views because he stated that he got angry watching events in Syria on television.

It is remarkable that the British Medical Association (BMA), the medical Royal Colleges and above all the General Medical Council (GMC) have to my knowledge not said a word. When I contacted the GMC to confirm this, I was told that the GMC had no formal position on Prevent. But they forwarded to me the Department of Health guidance on the duty of healthcare professionals *via-à-vis* Prevent, so presumably they are endorsing it.^1^ For a statutory body whose raison d'être is medical ethics, this is a dereliction of its core duty.

Teachers, university lecturers and others in the public sector are also being compelled to do the same thing. For civil liberties this is an ominous development within UK society, of a piece with the era of McCarthyism in the USA of the 1950s. It will end as badly as that era ended. The advocacy organisation CAGE has described such policies as consistent with a slide towards a ‘cradle to grave police state’.^2^ In July 2015 CAGE led and organised a joint statement opposing the Prevent strategy, with a letter published in The Independent newspaper signed by over 200 academics, activists, legal and medical professionals.^3^ The guidance given in the UK Prevent programme sets out a duty to prevent individuals from being drawn into terrorism, but clarifies that this means not only violent extremism but also non-violent extremism (i.e. thought and speech crimes). It is interesting that at least on paper the US Department of Homeland Security does not go so far, describing their programme as one which ‘focusses not on radical thought or speech but instead on preventing violent attacks’.^4^ Psychiatrists may be viewed as having particular access to a person's intimate thoughts and perceptions, so there is a particular challenge here for our specialty.

In my view, the Prevent workshops raise a significant medical ethical issue for doctors and should be a matter for the GMC to address. I have been canvassing psychiatric trainees and consultants in my trust. Many have expressed unease and are responsive to the idea that they would say no to these workshops.

Last, speaking as a citizen rather than just as a doctor, we need to understand how this situation has arisen, and why the security of the UK population might be better protected if the UK government reviewed their policies and alliances in the Middle East. A poll commissioned by *The Independent* found that 64% of people believed that Britain would be safer from a terrorist attack if the country had not been involved in conflicts in Iraq and Afghanistan.^5^ This region has over decades had to endure the unbridled application of Western power undeterred by the human and social destruction and dislocation this has caused. The rights and felt priorities of the civilian populations of the region, even in their millions, have weighed little on the scales by which the USA, the UK and Israel in particular have measured their interests. We know from multiple sources that well over 1 million people would be alive today if ‘we’ had not illegally invaded Iraq in 2003.^6^ What do these deaths weigh?
